# How much vocabulary is needed for comprehension of video lectures in MOOCs: A corpus-based study

**DOI:** 10.3389/fpsyg.2022.992638

**Published:** 2022-09-29

**Authors:** Ismail Xodabande, Hourieh Ebrahimi, Sedigheh Karimpour

**Affiliations:** ^1^Department of Foreign Languages, Kharazmi University, Tehran, Iran; ^2^Department of Foreign Languages, Islamic Azad University, Tehran, Iran; ^3^Department of English Language, Mazandaran University of Medical Sciences, Sari, Iran

**Keywords:** MOOCs, lexical coverage, lexical profile, vocabulary, corpus linguistics, word lists, video lectures, self-regulated learning

## Abstract

Over the past years, Massive Open Online Courses (MOOCs) have emerged as new competitive advantages in the digital economy of higher education globally. Accordingly, an increasing number of individuals are attracted to these new learning environments for developing their knowledge and skills in a variety of subject areas. Despite these developments, research on linguistic features of MOOCs lectures as the main mediums for delivering the course contents remained limited. To address this gap, the present study analyzed a corpus of MOOCs lectures with around 4.45 million words to determine the size of vocabulary knowledge needed for 95 and 98% coverages. The findings revealed that sufficient coverage of the course contents requires knowledge of the 5,000 most frequent words in English. Nonetheless, achieving adequate coverage level requires a much larger vocabulary size of around 9,000 most frequent words in English. The study also found that widely used word lists for general and academic vocabulary (i.e., the GSL/AWL) fail to support MOOCs learners with sufficient vocabulary knowledge for adequate lexical coverage. Based on these findings, the study draws a number of implications for preparing non-native English speakers to use MOOCs effectively and setting research-informed vocabulary learning goals in instructional programs and materials.

## Introduction

The last decade has witnessed a significant increase in the number of Massive Open Online Courses (MOOCs) as a competitive advantage in the digital economy of higher education (Guerrero et al., 2021). In this regard, it has been estimated that about 950 institutions are offering such courses for a large number of individuals around the world (Shah, 2020). Being publicly available *via* internet technology, these distance learning platforms provide participants with various affordances for knowledge development in different subject areas, while giving them the opportunity to decide on their learning pace, place and time (Otto et al., 2018; Fischer et al., 2020; Castaño-Muñoz and Rodrigues, 2021). Additionally, MOOCs are different from traditional formal systems as some gate-keeping requirements including educational background, previous accreditation, and fees are not compulsory for the participants. However, despite these attractive features, learners need to be autonomous in dealing with the content of the courses (Alonso-Mencía et al., 2020), which means that they mostly have to rely on their own abilities in self-directed learning mode (Zhu, 2021). As a large proportion of learning materials are delivered through video lectures in English, understanding these lectures is essential for successful participation in MOOCs.

Previous research shows that academic discourse in general might be challenging for most non-native speakers of English (Hyland, 2009; Dang, 2022), and insufficient vocabulary knowledge is among the important factors that contributes to inadequate comprehension of spoken academic English (Evans and Morrison, 2011; Dang and Webb, 2014). Moreover, although vocabulary demands of academic lectures has been investigated in the literature (Dang and Webb, 2014; Dang et al., 2017), a recent study revealed that MOOC lectures are generally different from traditional university lectures, as they are more abstract, non-narrative, highly informational, low in persuasion, explicitly referential, and formally planned (Yu, 2021). Consequently, given the paucity of research into linguistic features of MOOC lectures, it is not clear how much vocabulary is needed for understanding the content presented in such videos. To address this gap, the present study analyzed a large corpus of video lectures systematically collected from 194 MOOCs in the Coursera platform. More specifically, the study aimed to determine the level of English vocabulary knowledge needed for comprehending MOOC video lectures in English. Research in this area is significant as the findings can inform instructional programs in preparing learners for these emerging and rising educational environments. Furthermore, non-native speakers of English who are interested in lifelong learning with MOOCs might find the result helpful in setting their own vocabulary learning goals, which is in line with supporting and facilitating autonomous learning through MOOCs (Zhu, 2022).

## Literature review

In studying the relationship between vocabulary knowledge and comprehension, finding out the number of words that the readers (or listeners) should know for reasonable understanding of the text is a fundamental consideration (Laufer, 2020). Accordingly, lexical coverage, which is operationalized as the percentage of known words in a given text has been employed extensively in determining the vocabulary size needed for the comprehension of written or spoken discourse (Rodgers and Webb, 2016; Nurmukhamedov and Webb, 2019). Research in this area indicated that knowing 95–98% of the words in a text is necessary for having an acceptable comprehension level (Nation, 2006; Laufer and Ravenhorst-Kalovski, 2010; van Zeeland and Schmitt, 2012). More specifically, the 95% coverage has been regarded as the threshold for minimum comprehension, while the 98% figure is the optimal lexical coverage, which is necessary for adequate (or unassisted) understanding of texts (Laufer, 2020). Studies on lexical coverage also aim to determine the number of words corresponding to minimum or optimal thresholds (Schmitt et al., 2017). In this regard, it has been estimated that the 98% threshold in understanding written language requires knowing around 8,000 word families in English (Nation, 2006).

Over the past years, a growing number of studies investigated the vocabulary demands of spoken English (Webb and Rodgers, 2009a,b; Dang et al., 2017; Tegge, 2017; Nurmukhamedov and Webb, 2019; Nurmukhamedov and Sharakhimov, 2021; Dang, 2022; Ha, 2022; Phung and Ha, 2022). For example, to determine the vocabulary size needed to understand movies in English, Webb and Rodgers (2009a) analyzed the scripts of 318 movies with around 602 running hours and 2,841,887 words. The findings of the study revealed that the knowledge of the 3,000 most frequent word families is necessary for 95% lexical coverage, while for 98% coverage one must know at least 6,000 word families plus marginal words and proper names. Similar results were reported for vocabulary demand of TV programs in English, as 95% comprehension requires having knowledge of 3,000 most frequent vocabulary in English (Webb and Rodgers, 2009b). Nonetheless, TV programs are reported to be more lexically demanding, and 98% coverage needs knowledge of 7,000 word families (Webb and Rodgers, 2009b). In another study, Tegge (2017) investigated the lexical demands of English songs by analyzing two corpora with 408 and 635 pop songs, respectively. The source for the first corpus was US billboard charts, while the second corpus was made of songs selected by language teachers. The results pointed to considerably lower demand of song in terms of vocabulary knowledge compared to other written genres in English. With respect to the songs used by language teachers, the study found that knowledge of the 2,000 most frequent word families is sufficient for 95% coverage, however, 98% coverage required 4,000 words. The general picture provided by these studies shows that the knowledge of the most frequent vocabulary in English (i.e., 3,000 words) is essential for minimum comprehension threshold of movies and songs in English.

Previous studies also explored the vocabulary profile of spoken language used in academic and educational contexts. In this regard, Nurmukhamedov and Sharakhimov (2021) studied vocabulary demand of listening to English podcasts for language learning. Accordingly, it was found that the most frequent 3,000 word families plus proper nouns, marginal words, transparent compounds, and acronyms account for 96.75% of all words in the 1,137,163-word corpus compiled from the transcripts of 170 podcast episodes. In order to reach 98% coverage, podcasts listeners need the additional knowledge of 2,000 word families. Phung and Ha (2022) reported similar findings for the vocabulary knowledge needed for the listening section of the International English Language Testing System (IELTS). Moreover, the study indicated that the knowledge of the most frequent 2,000 words in English based on General Service List (GSL; West, 1953) and vocabulary items in the Academic Word List (AWL; Coxhead, 2000) is needed for 95% coverage. With respect to academic spoken English, Dang and Webb (2014) analyzed a corpus of 160 lectures and 39 seminars compiled from four disciplinary areas of the British Academic Spoken English (BASE) corpus. The study found that AWL accounted for only 4.41% of the corpus, which is considerably lower than the 10% coverage provided by this core academic word list in most academic discourse (Coxhead and Byrd, 2007). Additionally, the study found that 4,000 word families and proper nouns and marginal words provide around 96% coverage of academic spoken English, and knowledge of 8,000 words results in 98.00% coverage. Recently, Dang (2022) studied the lexical demand of conference presentations. The corpus contained 565,758 words developed from conference presentations in 20 academic subject areas, and the study found that the most frequent 3,000 words in English covered 97% of the presentations.

The expanding body of research related to lexical demands of spoken English in various contexts shows that vocabulary knowledge required to attain comprehension varies in different discourse types. This observation makes it necessary to analyze the lexical profile of MOOC lectures to establish the size of vocabulary needed for understanding the content presented in these emerging learning platforms. Additionally, without investigating the lexical profile of MOOC lectures, it is not easily possible to appreciate the value of corpus-based world lists for vocabulary learning and instruction. Accordingly, despite the wide spread application of the GSL (West, 1953) and the AWL (Coxhead, 2000) in addressing the vocabulary learning needs of language learners, the extent to which these lists support minimum and adequate comprehension thresholds in MOOC lectures remained unexplored. Moving along these lines, the current study addressed the following research questions:

1. How much vocabulary is needed for 95 for 98% coverages of MOOCs lectures?
2. What is the total coverage provided by the GSL and AWL in MOOCs lectures?

## Materials and methods

### Corpus

Following the widely used criteria for corpus building in terms of balance, representativeness, and size (McEnery and Hardie, 2011), transcripts of video lectures were systematically collected from 194 courses offered in the Coursera website for analyzing lexical coverage in the MOOCs. As for balance, the classification proposed by the Coursera was employed and courses were randomly selected from each of the 10 categories including (1) business, (2) computer sciences, (3) data science, (4) information technology, (5) health, (6) personal development, (7) physical science and engineering, (8) social science, (9) arts and humanities and (10) math and logic. One category, namely the language learning courses was excluded due to contents of the courses which were related to teaching different languages. The 10 sub-corpora were also balanced in terms of number of words, and each contained around 440,000 words with only small variation among the categories. With respect to representativeness, the study used Coursera platform which is one of the main MOOC providers (along with Udacity, edX, and FutureLearn). Additionally, as estimated in 2021, a large number of universities around the world (i.e., 150) offered around 4,000 MOOCs through Coursera website (de León, 2021). As for size, it has been argued that to ensure having a large sample of language use, a corpus should have millions of words (McEnery and Hardie, 2011). Accordingly, the corpus compiled for this study contained 4,448,604 words which is larger in size compared to the corpus used for developing the AWL (Coxhead, 2000).

### Corpus analysis software

In order to analyze the MOOCs corpus, the current study used AntWordProfiler (Anthony, 2021). The AntWordProfiler is a recently developed freeware for vocabulary profiling of texts. The tool works with a variety of input formats including Microsoft Word (.docx), TEXT (.txt), and PDF. After adding target corpus files into the AntWordProfiler, the software compares the loaded corpus against vocabulary lists and provides complete statistics for the words in the corpus. The GSL and the AWL are the default word lists in the software, however, users can add their own base lists for the analysis of the different corpora. In order to answer the research questions, the MOOCs corpus was profiled against British National Corpus (BNC)/Corpus of Contemporary American English (COCA) word lists (Nation, 2012), and GSL/AWL base lists.

## Results

The results of the lexical profiling of the MOOC lectures based on BNC/COCA lists (Nation, 2012) are summarized in Table 1. Accordingly, the most 1,000 common words in English cover a significant proportion of the words in the corpus (i.e., 80.48%). The second base list provided 7.29% coverage, and 2,000 most frequent words in English totally accounted for 87.77% of words in the corpus. The coverage for the third base list were lower the first two, and this list covered around 5% of the words in lectures. Taken together, the 3,000 most frequent word families in English based on BNC/COCA lists provided 92.85% coverage of the corpus, and there were around 13,000 word types (i.e., unique orthographic forms) identified by the corpus analysis software. Beyond these high frequency words, the coverage of the subsequent BNC/COCA lists dropped significantly. The analysis also indicated that knowledge of the 5,000 most frequent word families is needed for achieving 95% coverage. However, the 98% coverage requires much larger vocabulary size. Accordingly, considering the coverage of proper nouns, marginal words, transparent compounds, and acronyms (base list 31 to 34), this level of lexical coverage needs knowledge of 9,000 words. Additionally, around 12,103 word types that accounted for 0.86% of the corpus were beyond the base lists.

**Table 1:** The lexical profile of MOOC lectures based on BNC/COCA lists.

Statistics
Level	Token	Token%	CumToken%	Type	Group
1	3,580,032	80.48	80.48	4,632	999
2	324,152	7.29	87.77	4,249	997
3	226,104	5.08	92.85	4,218	1,000
4	64,462	1.45	94.3	2,883	991
5	37,601	0.85	95.15	2,252	948
6	26,006	0.58	95.73	1,916	920
7 to 25	84,511	1.89	97.62	9,145	6,484
31 to 34	67,541	1.52	99.14	4,983	4,459
0	38,195	0.86	100	12,103	12,103
Total	4,448,604			46,381	28,901

The results for the lexical profile of the MOOCs lectures based on GSL/AWL base lists are represented in Table 2. The findings indicated that the 1,000 most frequent words in English based on the GSL provided 81.42% of the running words in corpus. However, the coverage of the second GSL list was significantly lower compared to the first list, and the items in this list accounted for only 4.2% of the words in lectures. Moving to academic vocabulary, the AWL provided 5.32% coverage in the corpus. Overall, the GSL/AWL base lists accounted for 90.86% of the tokens, and 9,519 word types in the corpus. Around 9% of the words in MOOCs lectures were beyond vocabulary items in GSL/AWL.

**Table 2:** The lexical profile of MOOC lectures based on GSL/AWL lists.

Statistics
Level	Token	Token%	CumToken%	Type	Group
1^st^ GSL	3,622,025	81.42	81.42	3,794	998
2^nd^ GSL	183,362	4.12	85.54	3,069	970
AWL	236,547	5.32	90.86	2,656	569
0	406,670	9.14	100	36,862	36,862
TOTAL:	4,448,604			46,381	39,399

## Discussion and conclusion

The first research question was concerned with the amount of vocabulary needed for 95 and 98% coverages in MOOCs lectures. The findings indicated that for minimum comprehension threshold, MOOCs participants should know 5,000 most frequent word families in English based on BNC/COCA base lists (Nation, 2012). This vocabulary size amounts to around 13,000 word types. This finding is incongruent with earlier studies that investigated the lexical coverage in spoken discourse (Webb and Rodgers, 2009a,b; Tegge, 2017; Nurmukhamedov and Sharakhimov, 2021), and the present study indicates that MOOCs lectures are more demanding lexically. Additionally, data analysis revealed that even a larger vocabulary size in needed for adequate comprehension threshold. Accordingly, to achieve 98% coverage, a vocabulary size of 9,000 words seems necessary. This is partially in agreement with Dang and Webb (2014) who found that such level of lexical coverage in academic spoken English requires around 8,000 word families. Moreover, data analysis indicated that MOOCs need considerably larger vocabulary size compared to conference presentations (Dang, 2022). Overall, the study supports the earlier observations in terms of different nature of language used in MOOCs and highlights the need for more research language used in lectures (Yu, 2021).

The second research question explored the coverage of the GSL (West, 1953) and the AWL (Coxhead, 2000) in the corpus of MOOCs lectures. The findings indicated that general service vocabulary accounted for 85.54% of the corpus, and academic vocabulary provided around 5.32% coverage resulting in a cumulative coverage of 90.86% for the lists. The findings are congruent with Dang and Webb (2014) who reported 85.49% coverage for the GSL in British Academic Spoken English (BASE) corpus. However, the AWL accounted for more words in MOOCs lectures compared to 4.41% figure reported for BASE corpus (Dang and Webb, 2014). Findings of the current study also differ considerably with Phung and Ha (2022) that explored the lexical profile of listening test of IELTS, as the total coverage of the GSL/AWL in MOOCs lectures is significantly lower compared to 95% coverage in their corpus. These differences might have resulted from a number of factors. First, IELTS listening section is intended for testing English for academic studies and contains listening tasks in different levels of difficulty. This lowers the number of words needed for 95% comprehension. On the other hand, MOOCs are delivered by faculty members in a variety of disciplines and the language used in lectures is more authentic, abstract, and informational. Second, the BASE corpus analyzed by Dang and Webb (2014) was smaller in size compared to the corpus compiled for this study. The size of the corpus significantly impacts the occurrence of the words beyond high frequency vocabulary (Nation, 2016). Given that the AWL items by definition are those words beyond the GSL, this might have resulted in higher coverage. Additionally, the BASE corpus is based on lectures in two universities (i.e., University of Warwick and the University of Reading), while the corpus of MOOCs used in this study was based on courses offered by a large number of universities around the world. Although such diversity results in a more representative corpus, an inevitable outcome is having a less homogenous data base that impacts the lexical profile of the lectures.

The findings of the study have some implications for English language teachers and MOOCs learners. First, the study revealed that adequate understanding of lectures in English requires a large vocabulary size. This is specifically important for non-native speakers of English as their vocabulary growth and development takes considerable time spanning over several years (Webb and Chang, 2012; Rahmani et al., 2022; Xodabande et al., 2022; Zakian et al., 2022). With the growing appeal of MOOCs for delivering high-quality education for diverse populations and life-long learners around the world, there is an increasing need to prepare learners for dealing with the vocabulary demands of the video lectures. Among the various pedagogical interventions used for addressing vocabulary learning needs of foreign language learners, technology assisted vocabulary learning holds considerable potential (Lin and Lin, 2019; Xodabande and Atai, 2020; Hao et al., 2021). Accordingly, incorporating various technologies to augment vocabulary knowledge development might be a practical strategy for dealing with vocabulary demands of MOOCs. Second, the findings revealed that relying on well-established pedagogical word-lists such as the GSL and the AWL is not sufficient for vocabulary knowledge needed for MOOCs. Therefore, language teachers need to raise the awareness of the prospective MOOCs learners with respect to this issue and aim to addressing the vocabulary learning needs more systematically. Relatedly, the study shows that setting vocabulary learning goals based on BNC/COCA lists (Nation, 2012) which are developed using more contemporary and large corpora might result in more lexical coverage as the first three base lists accounted for around 93% of words in the lectures. Consequently, there is a need for developing vocabulary learning materials to teach vocabulary items in the BNC/COCA word lists. Third, although the vocabulary demand of MOOCs is higher relative to other spoken academic discourses, training learners in strategies to deal with unknown vocabulary in listening might contribute significantly to reducing the number of vocabulary items needed for sufficient comprehension. In this regard, previous research indicated that although 95% coverage is “relatively high and stable,” the 90% coverage might be also regarded “relatively high” for listening comprehension (van Zeeland and Schmitt, 2012, p. 474). Given that the 3,000 most frequent words cover a considerable proportion of the corpus; this vocabulary size might be regarded as the first step in preparing learners for self-regulated learning with MOOCs. Additionally, to ensure this relatively high coverage (i.e., 90%) MOOC learners might benefit from strategy training in terms of dealing with unknown vocabulary in context (Pavii Taka, 2008; Szudarski and Barclay, 2021).

The study had some limitations. First, only one platform is used for collecting video lectures in MOOCs. As there are some other main MOOCs providers (edX, Udacity, FutureLearn, etc.) that offer online courses from prestigious universities and institutions, the corpus analyzed in this study might not be representative of the contents offered in other platforms. This consideration should be accounted for in interpreting the results and there is a need for more research in this line of inquiry for having a more transparent picture of vocabulary demands of MOOCs. Moreover, although a large corpus was compiled and analyzed in this study, the size of the corpus significantly impacts corpus-based vocabulary studies (Nation, 2016). Accordingly, considering the difficulties associated with compiling spoken corpora (McEnery and Hardie, 2011), larger and more balanced corpora are needed to investigate the coverage provided by base lists beyond high-frequency vocabulary in MOOCs. Despite these limitations, the study provided valuable insights with respect to lexical profile of MOOCs and the size of vocabulary needed for understanding the contents. Future research might consider addressing these issues and also investigate the challenges faced by those participating in MOOCs in terms of insufficient vocabulary knowledge.

## Data availability statement

The original contributions presented in the study are included in the article/supplementary material, further inquiries can be directed to the corresponding author.

## Author contributions

All authors contributed to the design and implementation of the research, to the analysis of the results, and to the writing of the manuscript.

## Conflict of interest

The authors declare that the research was conducted in the absence of any commercial or financial relationships that could be construed as a potential conflict of interest.

## Publisher’s note

All claims expressed in this article are solely those of the authors and do not necessarily represent those of their affiliated organizations, or those of the publisher, the editors and the reviewers. Any product that may be evaluated in this article, or claim that may be made by its manufacturer, is not guaranteed or endorsed by the publisher.

## References

[ref1] Alonso-Mencía M. E. Alario-Hoyos C. Maldonado-Mahauad J. Estévez-Ayres I. Pérez-Sanagustín M. Delgado Kloos C. (2020). Self-regulated learning in MOOCs: lessons learned from a literature review. Educ. Rev. 72, 319–345. doi: 10.1080/00131911.2019.1566208

[ref2] Anthony L. (2021). AntWordProfiler (1.5.1w). Tokyo, Japan: Waseda University.

[ref3] Castaño-Muñoz J. Rodrigues M. (2021). Open to MOOCs? Evidence of their impact on labour market outcomes. Comput. Educ. 173:104289. doi: 10.1016/j.compedu.2021.104289, PMID: 34732973 PMC8407334

[ref4] Coxhead A. (2000). A new academic word list. TESOL Q. 34, 213–238. doi: 10.2307/3587951

[ref5] Coxhead A. Byrd P. (2007). Preparing writing teachers to teach the vocabulary and grammar of academic prose. J. Second. Lang. Writ. 16, 129–147. doi: 10.1016/j.jslw.2007.07.002

[ref6] Dang T. N. Y. (2022). A corpus-based study of vocabulary in conference presentations. J. Engl. Acad. Purp. 59:101144. doi: 10.1016/j.jeap.2022.101144

[ref7] Dang T. N. Y. Coxhead A. Webb S. (2017). The academic spoken word list. Lang. Learn. 67, 959–997. doi: 10.1111/lang.12253

[ref8] Dang T. N. Y. Webb S. (2014). The lexical profile of academic spoken English. Engl. Specif. Purp. 33, 66–76. doi: 10.1016/j.esp.2013.08.001

[ref17] de León R.. (2021). Coursera files for IPO amid online learning boom. Available at: https://www.cnbc.com/2021/03/05/coursera-files-for-ipo-amid-online-learning-boom-.html

[ref9] Evans S. Morrison B. (2011). Meeting the challenges of English-medium higher education: the first-year experience in Hong Kong. Engl. Specif. Purp. 30, 198–208. doi: 10.1016/j.esp.2011.01.001

[ref10] Fischer C. Pardos Z. A. Baker R. S. Williams J. J. Smyth P. Yu R.. (2020). Mining big data in education: affordances and challenges. Rev. Res. Educ. 44, 130–160. doi: 10.3102/0091732X20903304

[ref11] Guerrero M. Heaton S. Urbano D. (2021). Building universities’ intrapreneurial capabilities in the digital era: the role and impacts of massive open online courses (MOOCs). Technovation 99:102139. doi: 10.1016/j.technovation.2020.102139

[ref12] Ha H. T. (2022). Vocabulary demands of informal spoken English revisited: what does it take to understand movies, TV programs, and soap operas? Front. Psychol. 13:831684. doi: 10.3389/fpsyg.2022.831684, PMID: 35265018 PMC8899723

[ref13] Hao T. Wang Z. Ardasheva Y. (2021). Technology-assisted vocabulary learning for EFL learners: a meta-analysis. J. Res. Educ. Effect. 14, 645–667. doi: 10.1080/19345747.2021.1917028

[ref14] Hyland K. (2009). Academic discourse: English in a global context. London: Bloomsbury.

[ref15] Laufer B. (2020). Lexical coverages, inferencing unknown words and Reading comprehension: how are they related? TESOL Q. 54, 1076–1085. doi: 10.1002/tesq.3004

[ref16] Laufer B. Ravenhorst-Kalovski G. C. (2010). Lexical threshold revisited: lexical text coverage, learners’ vocabulary size and reading comprehension. Read. Foreign Lang. 22, 15–30.

[ref18] Lin J.-J. Lin H. (2019). Mobile-assisted ESL/EFL vocabulary learning: a systematic review and meta-analysis. Comput. Assist. Lang. Learn. 32, 878–919. doi: 10.1080/09588221.2018.1541359

[ref19] McEnery T. Hardie A. (2011). Corpus linguistics: method, theory and practice. Cambridge: Cambridge University Press.

[ref20] Nation I. S. P. (2006). How large a vocabulary is needed for Reading and listening? Can. Mod. Lang. Rev. 63, 59–82. doi: 10.1353/cml.2006.0049

[ref21] Nation I. S. P. (2012). The BNC/COCA word family lists. Available at: https://www.victoria.ac.nz/__data/assets/pdf_file/0004/1689349/Information-on-the-BNC_COCA-word-family-lists-20180705.pdf (Accessed September 22, 2022).

[ref22] Nation I. S. P. (2016). Making and using word lists for language learning and testing. Amsterdam: John Benjamins Publishing Company.

[ref23] Nurmukhamedov U. Sharakhimov S. (2021). Corpus-based vocabulary analysis of English podcasts. RELC J. 0033688220979315:003368822097931. doi: 10.1177/0033688220979315

[ref24] Nurmukhamedov U. Webb S. (2019). Lexical coverage and profiling. Lang. Teach. 52, 188–200. doi: 10.1017/S0261444819000028

[ref25] Otto D. Bollmann A. Becker S. Sander K. (2018). It’s the learning, stupid! Discussing the role of learning outcomes in MOOCs. Open Learn. 33, 203–220. doi: 10.1080/02680513.2018.1486183

[ref26] Pavii Taka V. (2008). Vocabulary learning strategies and foreign language acquisition. Clevedon: Multilingual Matters.

[ref27] Phung D. H. Ha H. T. (2022). Vocabulary demands of the IELTS listening test: an in-depth analysis. SAGE Open 12:215824402210799. doi: 10.1177/21582440221079934

[ref28] Rahmani A. Asadi V. Xodabande I. (2022). Using Mobile devices for vocabulary learning outside the classroom: improving the English as foreign language learners’ knowledge of high-frequency words. Front. Psychol. 13:899885. doi: 10.3389/fpsyg.2022.899885, PMID: 35814053 PMC9263603

[ref29] Rodgers M. P. H. Webb S. (2016). “Listening to lectures,” in The Routledge handbook of English for academic purposes. eds. Hyland K. Shaw P. (New York: Routledge), 165–176.

[ref30] Schmitt N. Cobb T. Horst M. Schmitt D. (2017). How much vocabulary is needed to use English? Replication of van Zeeland & Schmitt (2012), Nation (2006) and Cobb (2007). Lang. Teach. 50, 212–226. doi: 10.1017/S0261444815000075

[ref31] Shah D. (2020). The second year of the MOOC: A review of MOOC stats and trends in 2020. Available at: https://www.classcentral.com/report/the-second-year-of-the-mooc/ (Accessed September 22, 2022).

[ref32] Szudarski P. Barclay S. (eds.). (2021). Vocabulary theory, Patterning and Teaching. Multilingual Matters. doi: 10.21832/9781788923750

[ref33] Tegge F. (2017). The lexical coverage of popular songs in English language teaching. System 67, 87–98. doi: 10.1016/j.system.2017.04.016

[ref34] van Zeeland H. Schmitt N. (2012). Lexical coverage in L1 and L2 listening comprehension: the same or different from Reading comprehension? Appl. Linguis. 34, 457–479. doi: 10.1093/applin/ams074

[ref35] Webb S. Chang A. C.-S. (2012). Second language vocabulary growth. RELC J. 43, 113–126. doi: 10.1177/0033688212439367

[ref36] Webb S. Rodgers M. P. H. (2009a). The lexical coverage of movies. Appl. Linguis. 30, 407–427. doi: 10.1093/applin/amp010

[ref37] Webb S. Rodgers M. P. H. (2009b). Vocabulary demands of television programs. Lang. Learn. 59, 335–366. doi: 10.1111/j.1467-9922.2009.00509.x

[ref38] West M. (1953). A general service list of English words. Longman, Green & Co.

[ref39] Xodabande I. Atai M. R. (2020). Using mobile applications for self-directed learning of academic vocabulary among university students. Open Learn. 1–18. doi: 10.1080/02680513.2020.1847061

[ref40] Xodabande I. Pourhassan A. (Aydin), & Valizadeh, M. (2022). Self-directed learning of core vocabulary in English by EFL learners: comparing the outcomes from paper and mobile application flashcards. Comput. Educ. J. 9, 93–111. doi: 10.1007/S40692-021-00197-6

[ref41] Yu X. (2021). A multi-dimensional analysis of English-medium massive open online courses (MOOCs) video lectures in China. J. Engl. Acad. Purp. 55:101079. doi: 10.1016/j.jeap.2021.101079

[ref42] Zakian M. Xodabande I. Valizadeh M. Yousefvand M. (2022). Out-of-the-classroom learning of English vocabulary by EFL learners: investigating the effectiveness of mobile assisted learning with digital flashcards. Asian-Pacific J. Sec. Foreign Langu. Edu. 7, 1–16. doi: 10.1186/s40862-022-00143-8

[ref43] Zhu M. (2021). Enhancing MOOC learners’ skills for self-directed learning. Dist. Educ. 42, 441–460. doi: 10.1080/01587919.2021.1956302

[ref44] Zhu M. (2022). Designing and delivering MOOCs to motivate participants for self-directed learning. Open Learn. 1–20, 1–20. doi: 10.1080/02680513.2022.2026213

